# Spatio‐temporal metapopulation trends: The coconut crabs of Zanzibar

**DOI:** 10.1002/ece3.70168

**Published:** 2024-08-27

**Authors:** Rahel Sollmann, Tim Caro

**Affiliations:** ^1^ Department of Ecological Dynamics Leibniz Institute for Zoo and Wildlife Research Berlin Germany; ^2^ School of Biological Sciences University of Bristol Bristol UK

**Keywords:** *Birgus latro*, monitoring, Pemba, population dynamics, sex ratio, weight

## Abstract

Species experience a variety of environmental and anthropogenic conditions across their ranges leading to spatial variation in population dynamics. Understanding population dynamics under different conditions is important but it is challenging to allocate limited effort to spatial and temporal subpopulation monitoring. Using GLMMs, we analyze survey data of a metapopulation of coconut crabs spanning 7 years and 15 sites in and near the Pemba archipelago, Zanzibar, to estimate trends in population size (based on catch per unit effort), weight and sex ratio at the meta‐ and subpopulation level and investigate anthropogenic drivers of these trends. We found that the overall metapopulation has remained stable in terms of size and composition over the survey period, but observed diverging trends in population size and sex ratio at some subpopulations. Formal protection of sites was associated with positive population trends. Of nine sites for which we could estimate site‐specific trends, three showed increasing and two decreasing trends, whereas four sites had stable subpopulations. Although anthropogenic factors affected the average weight, and the incidence of small and large individuals, we found no temporal trends in any weight‐related measures. Furthermore, there were no apparent patterns between weight‐related measures and subpopulation trends. The metapopulation was biased toward males, and exploitation appeared to be associated with declining trends in the proportion of females, likely an artifact of a strong decline in the proportion of females in one of only two exploited sites in the dataset. Educational campaigns implemented in 2020 at six sites were not related to higher population sizes in later surveys. The variable trends in subpopulation sizes and composition highlight the need for spatially replicated monitoring in metapopulations. The analyses further provide a detailed baseline for future subpopulation studies of this vulnerable species in one of its last remaining metapopulations in the Western Indian Ocean.

## 1 INTRODUCTION

Species naturally tend to experience a variety of environmental conditions across their ranges leading to spatial variation in population dynamics (Kareiva, 1990). In the age of global change, as animal populations decline globally (Brodie et al., 2021; Houlahan et al., 2000), many formerly continuous populations are becoming increasingly fragmented. The resulting subpopulations may inhabit distinct habitats, potentially ranging from fully protected areas to areas with high levels of anthropogenic impacts (depending on species tolerance/adaptability and geographic location). Subpopulation size can also affect their dynamics, with smaller populations at higher risk of decline due to stochastic events and inbreeding (Gilpin & Soulé, 1986). Moreover, when multiple subpopulations are connected to each other via dispersal of individuals (i.e., form a metapopulation), their level of connectedness and dynamics of nearby subpopulations can further shape local population trends (Gundersen et al., 2001; Hanski, 1998).

Although conservation actions often take place on a local scale (e.g., management decisions are made for a particular protected area; Wiens & Bachelet, 2010), for most species of conservation concern it is understood that safeguarding them requires a broader perspective. Individual protected areas, for example, are often not large enough to sustain demographically and genetically viable populations, requiring a landscape/regional scale approach to conservation (Margules & Pressey, 2000; Noss & Daly, 2006). In this context, it is vital to monitor subpopulations across environmental gradients, as cherry picking certain “flagship” sites such as populations living in national parks, or suffering certain site‐specific anthropogenic pressures, can lead to erroneous conclusions about the population as a whole. Moreover, monitoring species at large spatial scales allows us to study drivers (or correlates) of spatio‐temporal variation (Lindenmayer et al., 2022), thereby moving from mere observational monitoring to being able to test ecological hypotheses and make predictions that can inform proactive interventions.

The coconut crab, *Birgus latro*, living on the Zanzibar archipelago in the Western Indian Ocean (Caro et al., 2024) represents a threatened metapopulation. Coconut crabs are the world's largest terrestrial arthropod reaching 4 kg in body mass and 1 m in leg span (Brown & Fielder, 1991; Drew et al., 2010). Following a short larval stage at sea, individuals move on to land, where very young stages carry an empty mollusk shell, and generally remain in coastal scrub or forest for the rest of their lives (Brown & Fielder, 1991). As a result, adults cannot move between discrete subpopulations, but larvae can, so that adult mortality is site‐specific but recruitment is metapopulation specific. Coconut crabs are almost immune to natural predation but are threatened by anthropogenic exploitation for food and habitat destruction owing to agricultural expansion and coastal housing and hotel development (Cumberlidge et al., 2022). Now, virtually extirpated from continental landmasses, coconut crabs are mostly found on island archipelagos across the Indian Ocean and Western Pacific Ocean, such as Zanzibar, where they inhabit remaining patches of coastal rag forest. As a result of these threats, this species has recently been upgraded from “Data Deficient” to “Vulnerable” on the IUCN Red List (Cumberlidge, 2020).

Although the species has been in decline globally, next to nothing is known about population trends at local and regional scales, except for anecdotal evidence of declines (e.g., Widiyanti et al., 2016). Our objective was to characterize trends in population size and composition with respect to sex and size for the overall metapopulation, as well as individual subpopulations, of the Zanzibari coconut crab, and to identify anthropogenic drivers of such trends. Specifically, we imagined that human persecution would lead to a decline in larger individuals (particularly males that are larger than females) at some sites, but would reduce recruitment over time across the whole metapopulation since older larger individuals disproportionately contribute to recruitment (Sato & Suzuki, 2010). We also imagined that loss of coastal rag forest habitat to agriculture and building at unprotected sites could lead to a decline in large individuals through reduced adult survival, and/or small individuals through reduced recruitment, owing to increased competition for limited resources. We expected that both persecution and habitat loss would negatively affect population trends. Understanding which conditions benefit coconut crab populations can inform conservation and sustainable development in their last stronghold in the Western Indian Ocean. Beyond coconut crabs, our study highlights the need for spatially replicated monitoring for a fuller understanding of the dynamics and conservation status of fragmented populations.

## 2 METHODS

### 2.1 Field data collection

Research was principally conducted on the island of Pemba and its outlying islands in the United Republic of Zanzibar archipelago, across 29 sites but only 15 were visited repeatedly and only these 15 were used in this study. Pemba (988 km^2^, 14 of the analyzed sites) is an oceanic island lying 50 km east of the Tanzania mainland. The island is hilly with fertile soil and is dominated by small scale farming of cassava, tomatoes, bananas, with cloves (*Syzygium aromaticum*) as the cash crop, as well as seaweed. It has a large human population of ca. 543,000 people. Unguja (1666 km^2^, 1 analyzed site), the other main island in the Zanzibar archipelago, is a landbridge island lying 59 km to the south. The island is flat and relies very heavily on the tourism industry and fishing and considerable seaweed farming; c. 1,346,000 people live there according to the 2022 census.

**FIGURE 1:**
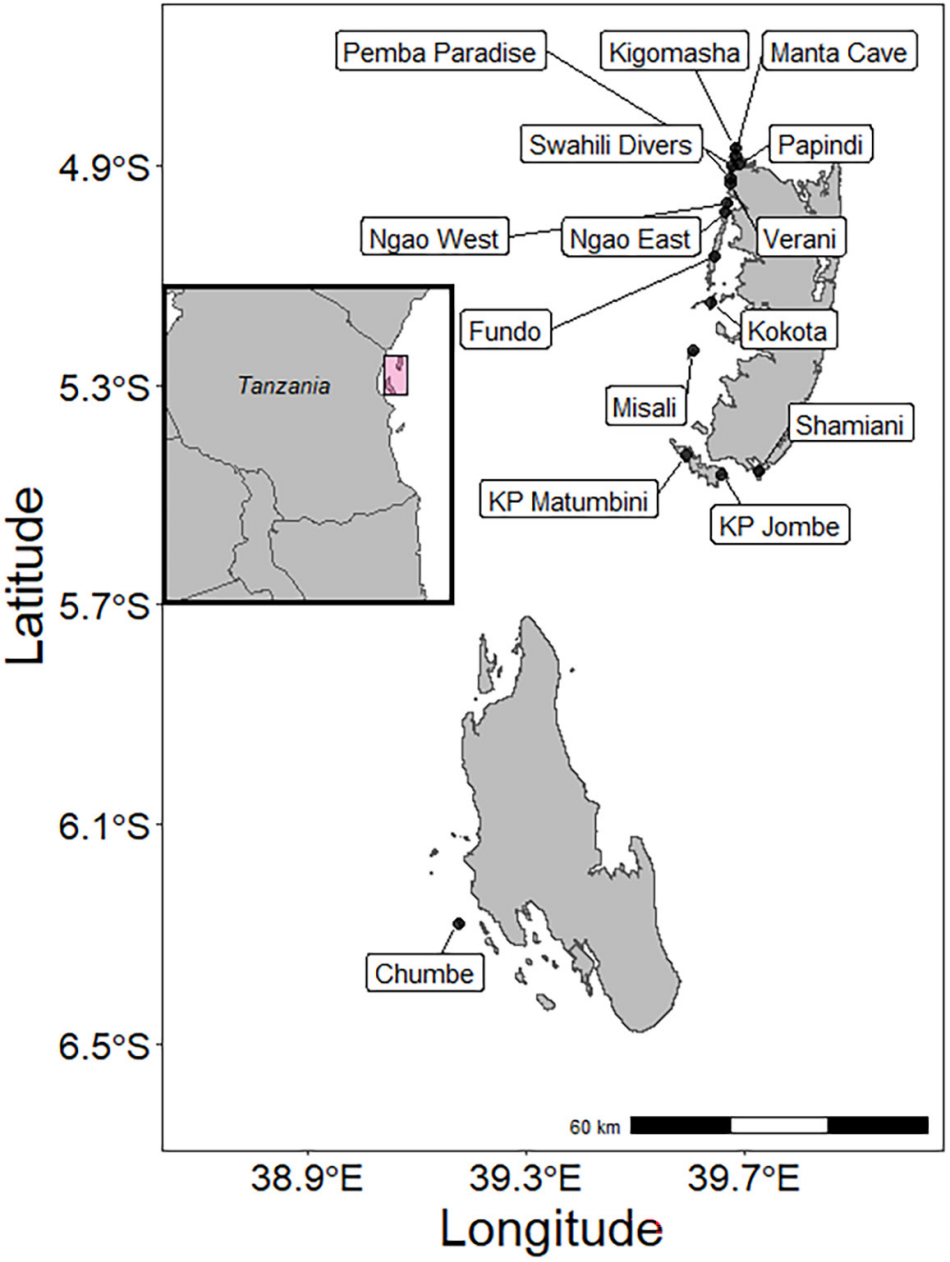
Sites at which coconut crabs were sampled between 2016 and 2023 on/near the Pemba archipelago, Zanzibar. Zanzibar is located off the east coast of Tanzania, East Africa, and is part of that nation.

We conducted searches of adult coconut crabs (defined as individuals that had lost their gastropod shell and were fully terrestrial) at all sites (Figure 1) between July 2016 and August 2023 (see Caro et al., 2024 for details on surveys). Survey periods were principally in the early (July to September) or the late dry season (December to January) to maximize the number of nights that could be spent in the field, but some sites were also surveyed in the wet season (March–June) in 2022 and 2023. Sampling sites were identified based on local information, and areas to search within those sites were chosen by fishermen or farmers familiar with the area and who usually took part in the surveys. Groups of between one and six persons (mean = 2.50 ± 1.16 persons; *N* = 179 nights) conducted crab surveys along transects or within search areas. Surveyors, spread out and equipped with torches, walked slowly starting between 19.15 h and 20.30 h and continued for 2–4.5 h. We recorded several measures of survey effort based on time the team spent in the field on a visit, time actively spent searching, and number of people searching (see Table S1).

Once detected, each crab was captured and placed in a bucket, weighed, sexed, and measured. Before release, it was photographed and marked with a uniquely assigned number using a white marker (Edding 950 industrial painter) lasting the length of a survey (maximum 3 months), so that subsequent recaptures could be identified. Surveys consisted of one or multiple visits (defined as one night searching) to the same site within a season. There were four types of surveys: (i) on Pemba mainland sites (Figure 1), usually repeated sampling occasions separated by an approximate minimum of a week; (ii) on islands off the coast of Pemba (where additional visits would be prohibitively difficult) with an assistant from the Department of Forestry, either one night or two consecutive nights; (iii) on Chumbe island up to 5 consecutive nights, with one survey consisting of two such bouts within 2 months; and (iv) on Misali and Chumbe islands by two students using repeat sampling occasions, approximately twice or once per week, respectively, and over a span of 3 and 11 weeks respectively (note that these students surveyed multiple locations, but only data from locations corresponding to earlier surveys were included here).

### 2.2 Data analysis

To characterize population dynamics for the metapopulation overall, we analyzed survey data from 15 sites or subpopulations, surveyed repeatedly (between 2 and 10 surveys; Table 1, Figure 1) during the overall study period between 2016 and 2023. Models fit to the complete dataset always contained a site random effect to account for repeated surveys at the same sites. We further tested the effect of several site‐level predictors on the baseline and trends in response variables (for details, see below).

**TABLE 1:** Overview of sites on/near Pemba surveyed for coconut crabs, with number of surveys, total number of nights, years spanned by surveys, binary predictor values (Agri = nearby agriculture, Protect = government protection, Exploit = exploitation, Village = nearby village, Hotel = nearby hotel) for models fit to data, and start year of education campaign (if applicable).

Site	Surveys	Nights	Years	Agri	Protect	Exploit	Village	Hotel	Campaign
Chumbe^a^	6	39	2017–2023	0	1	0	0	1	/
Fundo^a^	6	12	2016–2023	1	0	0	0	0	2020
Kigomasha^a^	9	23	2016–2023	1	0	0	1	0	2020
Kokota^a^	4	4	2019–2023	1	0	0	0	0	2020
KP Jombe^a^	5	5	2017–2023	0	0	1	0	0	2022
KP Matumbini^a^	5	7	2017–2023	0	0	1	0	0	2022
Manta Cave	3	5	2022–2023	1	0	0	1	0	/
Misali^a^	6	17	2016–2023	0	1	0	0	0	2020
Ngao East	2	2	2022–2023	1	0	0	0	0	2022
Ngao West	2	2	2019–2023	1	0	0	0	0	2022
Papindi	2	3	2017–2018	0	0	0	0	0	/
Pemba Paradise	3	4	2022–2023	1	0	0	1	1	/
Shamiani	3	6	2022–2023	1	0	0	0	1	2022
Swahili Divers^a^	10	31	2016–2023	1	0	0	1	1	2020
Verani^a^	7	19	2017–2023	1	0	0	1	1	2020

^a^ Data‐rich sites.

Because sampling at 6 of these subpopulations was limited to at most three surveys spanning few years (Table 1), thus providing little information about trends, we also analyzed a subset of 9 sites for which sampling spanned most of the study period (referred to as data‐rich sites). Our goal with this analysis was to characterize population dynamics at these well‐surveyed sites and discuss these in a site‐specific context. Models for this data subset therefore contained a fixed site effect and a site‐trend interaction (i.e., estimate trends independently for each site). All models were fit in R ver. 4.2.1 (R Core Team, 2022) using the package glmmTMB ver. 1.1.7 (Brooks et al., 2017).

### 2.3 Population trends

To investigate population trends, we analyzed the number of crabs captured per visit with a Poisson mixed model, using field effort as an offset. This is akin to analyzing catch per unit effort (CPUE) and does not allow estimating absolute abundance. However, as long as capture probability of crabs remains constant over time, or factors that affect capture probability can be modeled, analysis of CPUE can provide information on trends.

Because some surveys consisted of multiple nightly visits, and because we consider population size to be stable during a survey, we added a survey random effect nested within site to the site‐level random effect. We compared models with this random effects structure and three different measures of field effort as offset using Akaike's Information Criterion (AIC, Burnham & Anderson, 2001; see Table S1 for details). The top model (>160 units of AIC from the second model) contained the amount of time in the field during a given visit and we used this effort measure in all subsequent models.

Second, we added a year‐season (wet or dry) random effect to the model to explore synchronous variation in crab populations over time across all sites. The model with this year‐season random effect had less AIC support than the model without (ΔAIC = 1.91) and we moved forward without this random effect.

Next, we tested for an effect of several visit‐level variables thought to influence capture probability of crabs, by building single‐covariate models. Specifically, we tested: percent moon (0 to 100% taken from the Moonrise, moonset, and phase calendar https://www.timeanddate.com/moon/@150733); binary moon phase (with 1 corresponding to moon phase nights 12–16, i.e., near/full moon, and 0 corresponding to all other moon phases), because local knowledge—and possibly literature (Amesbury, 1980) suggests less crab activity with light; precipitation the day of the visit (both in mm and binary yes/no) and total precipitation in the week preceding the visit (in mm), because local knowledge suggests more activity with rain. We obtained precipitation data from CHIRPS (https://www.chc.ucsb.edu/data/chirps) which provides daily rainfall at the level of the ward (*shehia* in Kiswahili). We assigned each site to the *shehia* in which it was located, or, if data were missing for a given *shehia* (because >50% of the grid cells making up a *shehia* were over the ocean, in which case CHIRPS does not provide an estimate), the closest *shehia* with available data. We compared these single‐covariate models via AIC. The model without any visit covariate was within 2 ΔAIC of the top model (see Results and Table S2). This suggests that none of the covariates was particularly important in predicting crab capture numbers. We therefore moved forward retaining only the variable in the top model, binary moon phase, in subsequent CPUE models.

Finally, to estimate population trends, we added a covariate measuring time elapsed since the first survey (in years) to the base model. We calculated this covariate at the survey mid‐point and used the same survey‐level value for all visits within a survey. We calculated the covariate relative to the first survey at a given site, to account for the fact that the start of sampling varied across sites (Table 1). We refrained from more complex trends (e.g., quadratic or higher order polynomials) because of the shortness of the time series (approximately 7 years max). Because coconut crabs are long‐lived (Brown & Fielder, 1991), we did not consider additional factors such as seasonality to affect their survey‐specific numbers. We refer to the model with binary moon phase and trend as the base trend model.

To explore drivers of population trends in the full dataset, we added three binary site‐level predictors to the base model, one at a time owing to the small sample size at the site level (Table 1). We chose these three predictors as they have been found to affect CPUE in two previous analyses (Caro et al., 2021, 2024): the presence of agriculture (with mixed evidence as to the direction of the effect), government protection (affecting crab populations positively), and the presence of nearby villages (affecting crab populations negatively). Although other variables could conceivably affect crab populations and trends, we limited ourselves to these predictors to reduce the risk of “detecting” spurious relationships between CPUE and predictors. We first ran three models adding each predictor as a main effect only to the base trend model, i.e., exploring its effect on baseline CPUE, and compared them with the base trend model. We retained the important predictors, which we defined as within 2 ΔAIC of the top model and >2 ΔAIC better than the base trend model. We report trend estimates under this best main effects model as a measure of an overall metapopulation trend. To investigate whether trend was affected by site‐level characteristics, we then added each predictor in turn to the best main effects model as an interaction effect with trend and compared these models with the best main effects model.

Finally, we fit a model with effort offset, moon phase and trend, site as fixed effect and a site‐trend interaction to the data subset of the nine data‐rich sites to estimate site‐specific trends. We derived population rates of change, λ, from coefficient estimates for the trend variable and used the delta method to calculate their standard errors and Wald confidence intervals. We classified populations into “growing” (λ >1.1), “stable” (λ between 0.9 and 1.1) and “declining” (λ <0.9).

### 2.4 Weights

Weight of coconut crabs is related to their age and reproductive potential (Sato & Suzuki, 2010), and we analyzed trends in weight to gauge demographic “health” of subpopulations and the metapopulation over time. A decline in weight may indicate size‐biased offtake (Sato & Yoseda, 2010) or low adult survival due to other factors (e.g., loss of suitable habitat). An increase in weight may indicate a loss of recruitment of young. We excluded two sites/subpopulations (Ngao East, Manta Cave) from the weight analysis because they only had one survey that yielded some captures, and thus cannot contribute to weight trend estimation. Individuals recaptured within a survey were not weighed for a second time, but if recaptured in a different survey, their new weight was recorded. Because individuals were not identified for the 2023 surveys, we did not account for individual ID in our weight analysis. Such recaptures across surveys were rare, accounting for 57 of the 769 data points collected before 2023, and therefore, ignoring this lack of independence should not affect model results. Weights from the two student‐led surveys on Chumbe and Misali (dry and wet season 2022 respectively) were excluded to ensure consistency in weight measurements.

We analyzed square‐root transformed weights (kg) with linear mixed models, with site‐level random intercepts and a site‐specific error variance (analogous to Caro et al., 2024), including sex as a fixed effect to control for size dimorphism, and time since survey start (see CPUE models) to estimate a linear annual trend.

Because the species is so slow growing (Fletcher et al., 1990) and average weight may not change much if losses of individuals occur at the extremes of the weight distribution, we also analyzed the incidence of small and large individuals. We calculated the 25th and 75th percentile of all weights measured across all subpopulations and surveys and classified individuals as small or large if they fell below the 25th or above the 75th percentile, respectively. We analyzed incidence of small and large individuals as two separate logistic regressions, with site‐level random intercepts, sex as a fixed effect and an annual trend.

For all three weight measures, we built upon the above‐described base models following a similar procedure as for the CPUE models. We first tested the main effect of three binary covariates previously identified to affect crab weight (Caro et al., 2021, 2024), namely, government protection, the presence of hotels (both affecting weights positively) and exploitation (affecting weights negatively; Table 1). We retained the most important main effect based on AIC; and then tested for interactions of the same three predictors with the trend variable.

Because model results provided no evidence of trends in weights or incidence of small or large individuals (see Section 3), we refrained from a separate analysis of the 9 data‐rich sites.

### 2.5 Proportion of females

Females may be limiting in male‐biased populations such as this (2:1) and other coconut crab populations (Appoo et al., 2021). To investigate potential changes in sex composition over time, we analyzed the incidence of females, treating sex as a binary response variable. As for weights, we removed the two sites for which only a single survey yielded captures. Similar to the other analyses, the logistic regression contained a site random intercept and annual trend. As we had no previous studies to draw from to determine potential predictors of the incidence of females, we explored the presence of agriculture, protection and exploitation, as these were important predictors of CPUE and/or weight in this study (see Section 3). We followed the same model building/selection strategy as for CPUE and weight, testing for important main effects first, then for interactions with the trend variable.

In addition, we fit a logistic regression with fixed site effect and a site‐trend interaction to data from the 9 data‐rich sites to estimate site‐specific trends in the proportion of females.

### 2.6 Model fit

For all response variables and for both the full dataset and the subset of data‐rich sites where applicable, we evaluated fit of the final trend model using residual plots (Figures S1–S10) from the DHARMa package v. 0.4.6 (Hartig, 2022a). For the full data set of CPUE, residual plots suggested mild lack of fit of the final model; typical strategies to address this issue (using a negative binomial distribution; adding random variation; testing for zero‐inflation) failed to address lack of fit (Figures S1–S4). Because residuals only suggested mild heteroscedasticity, we decided to keep the final model, but caution that standard errors of estimates may be overly conservative. The same held true for the incidence of large individuals (Figure S8), where lack of fit was even less pronounced and significance likely a result of the relatively large sample size. There was no lack of fit for the incidence of small individuals (Figure S7). For all other response variables and datasets, the best trend model generally fit the data well, but there were some patterns in residuals (Figures S5, S6, S9 and S10). Such patterns can be the result of using random effects (Hartig, 2022b); visually, these patterns were rather weak. We therefore concluded that models fit data adequately for inference.

### 2.7 Additional information

**FIGURE 2:**
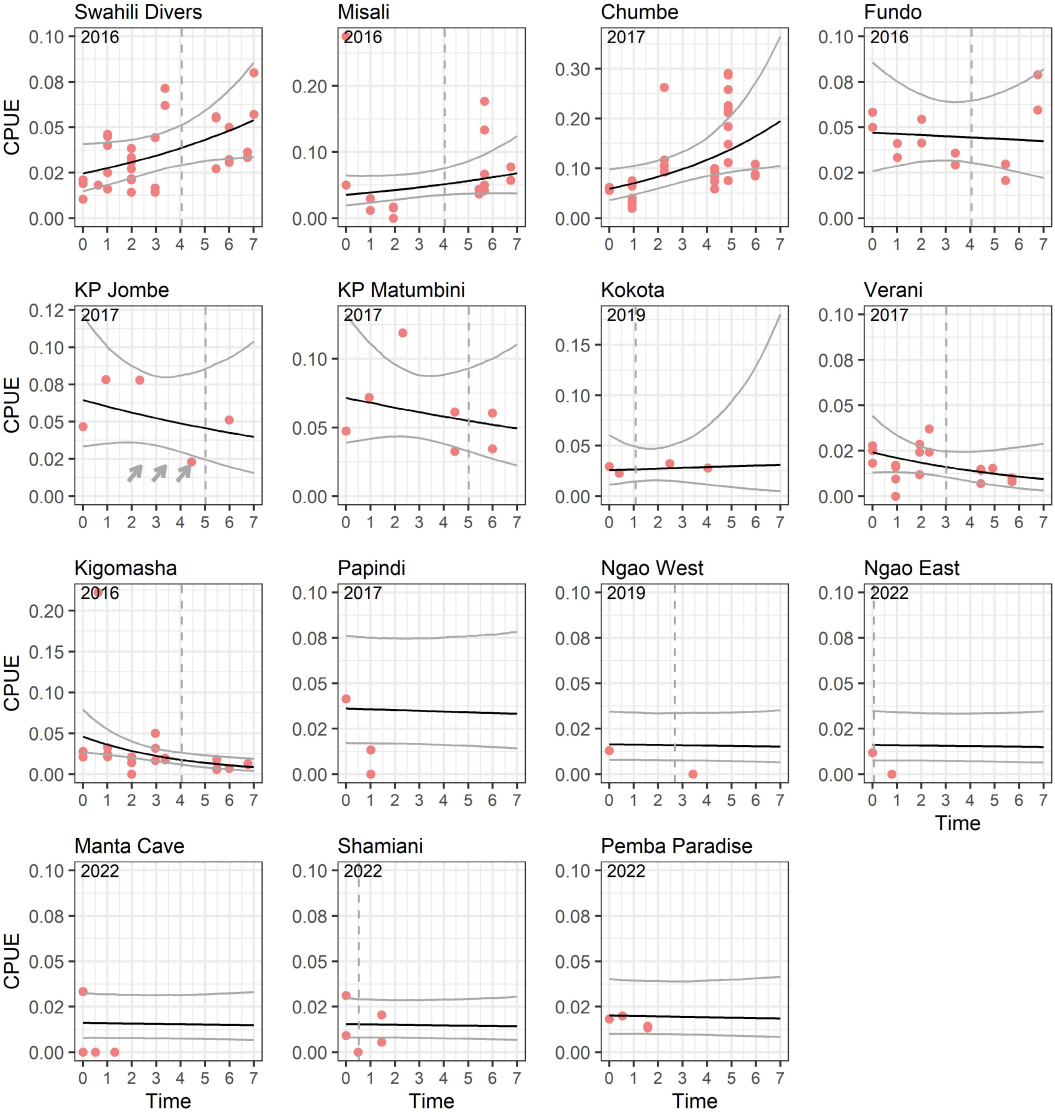
Catch per unit effort (CPUE; crabs captured per minute in the field) over time at 15 sites in/near Pemba. Year 0 refers to the first year a site was surveyed (shown on top of each subplot). Black lines show estimated trends in CPUE from a Poisson GLMM; gray lines show 95% confidence intervals. For sites from Swahili Divers to Kigomasha, the model contained a fixed site specific intercept and trend; for remaining sites, the model contained a random site intercept, the effect of agriculture on baseline CPUE (only Papindi had no agriculture) and the effect of protection (all unprotected) on trend. Red dots show CPUE per visit (possibly with multiple visits per survey) for all surveys to a site. Dashed vertical lines show timing of education campaign at site. Arrows (for KP Jombe) show timing of removal of crabs by the Department of Forestry. Note the different scales of the y axis.

To further contextualize results from our analyses, we compiled some additional information. First, we compiled dates of education campaigns implemented at 11 of the 15 sites by the Department of Forestry (Table 1) and added these to the results figure for the CPUE data (Figure 2). In brief, the campaigns consisted of putting up permanent large signboards at small ports, villages and schools at a site and in some cases giving a seminar to children or fishermen about the importance of coconut crab conservation at the same time. For six sites, these campaigns were implemented in September 2020. To test whether these campaigns had a positive effect on coconut crab populations, we subset the CPUE data to these 6 sites and fit an additional Poisson mixed model with the same structure as the model for data‐rich sites, but replacing the site‐specific trends with a binary main effect indicating whether a survey took place before or after the campaign (i.e., testing for a difference in CPUE before and after campaigns that is consistent across sites). For the remaining five sites, campaigns were implemented in September 2022 and we do not anticipate populations of a long‐lived species such as coconut crabs to show a response that soon.

Second, we compiled information on removal of crabs by the Department of Forestry for an annual agricultural fair from a single site, KP Jombe, and added these to the CPUE results figure.

Third, we compiled capture data collected by others on Chumbe (1999–2010) and Misali (2006–2018) during student projects from unpublished project reports. We compiled the total number of crabs captured, calculated sampling effort as the number of locations surveyed multiplied with the number of nights surveyed, and obtained CPUE by dividing number of crabs captured by effort. When the number of nights surveyed was not explicitly specified, we assumed that surveys took place throughout the study duration, which was between 2 and 3 weeks. It is important to note that this measure of CPUE is not comparable to that used in our analyses as a different measure of effort is used and as it hinges on untestable assumptions about effort when that was not recorded. Moreover, it does not account for visit‐level conditions such as moon phase or rainfall; neither does it account for the fact that projects surveyed different locations, some used bait, whereas others did not, and locations were spread across different spatial extents. Nonetheless we include it in Data S1 as a rough check on whether trends in numbers over time from these surveys correspond to trends in our more systematic surveys.

## 3 RESULTS

### 3.1 Population trends

Investigating visit level variables and their effect on crab CPUE, the top model contained an effect of binary moon phase, with lower CPUE during (near‐) full moon (*β*: −0.222, SE 0.119); models with similar support (within 2 ΔAIC) were the null model, and models with daily or weekly rain, but effects of rain were weak (Table S2).

The only important binary predictor of baseline CPUE (i.e., main effect) in the full dataset was the presence of agriculture (Table 2), with sites with agriculture having lower CPUE than sites without. This main effects model suggested a stable metapopulation of coconut crabs over the 7 years survey period (annual trend on the log scale: 0.028, SE 0.034).

**TABLE 2:** Model selection of binary site‐level predictors affecting coconut crab baseline catch per unit effort (CPUE; Effect = Main) and annual trend (Effect = Interaction) in CPUE.

Effect	Variable	ΔAIC	Beta	SE
Main	***Agriculture***	0	−0.982	0.259
Main	Protected	4.78	1.036	0.413
Main	NULL	8.07	/	/
Main	Village	8.44	−0.487	0.357
Interaction	***Protected***	0	0.143	0.067
Interaction	NULL	2.53	/	/
Interaction	Agriculture	3.31	−0.074	0.067
Interaction	Village	3.4	−0.064	0.06

*Note*: The “Main” base model (Variable = NULL) only has an annual trend (plus additional model structure accounting for study design and variation in capture probability); the “Interaction” base model is equal to the best “Main” model. Variables in top model are in bold and italic if base model is >2 **Δ**AIC from top model.

The only important binary predictor of population trends was government protection (Table 2), with populations in protected sites growing (annual trend: 0.130, SE 0.057), compared with stable populations at unprotected sites (annual trend: −0.012, SE 0.038).

The analysis of the data subset of 9 data‐rich sites showed that populations were growing at Swahili Divers (a mainland hotel area), Chumbe and Misali (two protected islands); populations were declining in Kigomasha (a Pemba mainland area) and Verani (a mainland non‐running hotel area); and populations appeared stable at four sites (Fundo and Kokota—inhabited islands, KP Jombe and KP Matumbini—uninhabited islands; Figure 2, Table S3).

### 3.2 Weights

**FIGURE 3:**
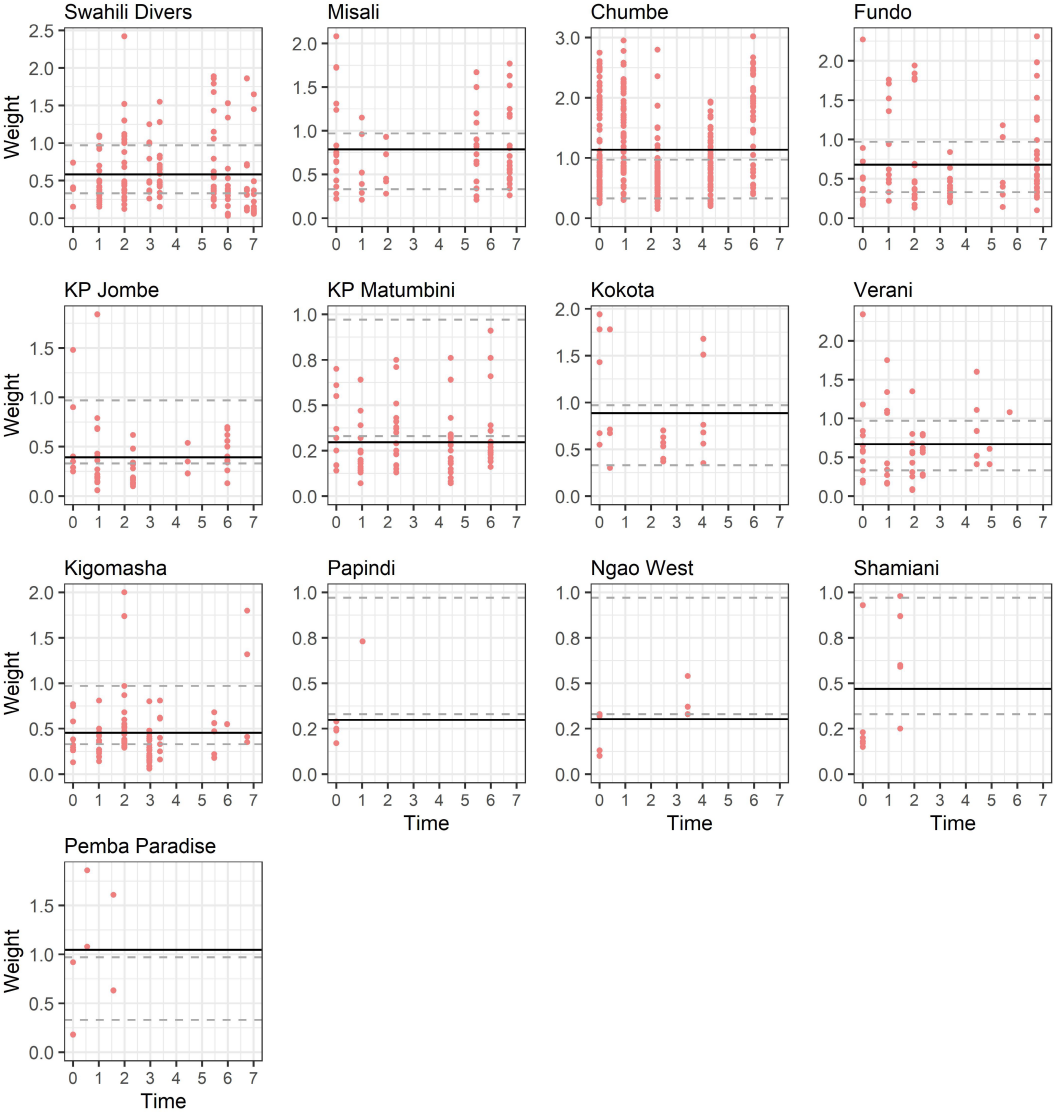
Weights (in kg) of coconut crabs caught over time at 13 sites in/near Pemba (two surveyed sites were excluded because they only yielded captures during a single survey). Year 0 refers to the first year a site was surveyed, not a calendar year. Weights are aggregated by survey, which may span several nights of sampling. Solid black line shows site‐specific mean weight across surveys; dashed gray lines indicate 25th and 75th percentile of all weights (across sites and surveys), used to define small and large individuals. Model suggested no trends in weights or incidence of large/small individuals; therefore no trends are plotted. Note the different scales of the y axis.

The full dataset included 932 weight measures (Figure 3), with 37% from females (mean weight 0.48 kg, SD 0.23) and 63% from males (mean 0.91 kg, SD 0.70).

Of the three binary main effects, both protection and exploitation were important. We therefore added a model to this selection step with both predictors, which emerged as the single top main effect model (Table 3a). This main effects model suggested average weight in the metapopulation of coconut crabs remained stable over the 7 years survey period (annual trend: 0.003, SE 0.004).

**TABLE 3:** Model selection of binary site‐level predictors affecting baseline values (Effect = Main) and annual trends (Effect = Interaction) in (a) (square root transformed) crab weight, (b) probability of being small, and (c) probability of being large.

	Effect	Variable	ΔAIC	Beta	SE
(a)					
	Main	***Protected + Exploited***	0	0.217 −0.175	0.064 0.064
	Main	Protected	3.51	0.25	0.084
	Main	Exploited	5.03	−0.222	0.094
	Main	Hotel	5.83	0.159	0.077
	Main	NULL	7.54	/	/
	Interaction	NULL	0	/	/
	Interaction	Hotel	1.1	0.007	0.008
	Interaction	Protected	1.53	−0.006	0.009
	Interaction	Exploited	1.75	0.004	0.007

(b)					
	Main	***Protected***	0	−2.079	0.663
	Main	Exploited	3.43	1.541	0.792
	Main	NULL	4.49	/	/
	Main	Hotel	4.7	−0.954	0.686
	Interaction	NULL	0	/	/
	Interaction	Hotel	0.35	0.103	0.081
	Interaction	Exploited	1.77	0.049	0.101
	Interaction	Protected	1.78	−0.048	0.103

(c)					
	Main	***Hotel***	0	1.832	0.76
	Main	Protected	1.89	1.866	0.933
	Main	NULL	3.22	/	/
	Interaction	Protected	0	0.057	0.087
	Interaction	NULL	0.31	/	/
	Interaction	Hotel	0.32	−0.029	0.088

*Note*: The “Main” base model (Variable = NULL) only has an annual trend (plus additional model structure accounting for study design and sex dimorphism); the “Interaction” base model is equal to the best main effects model. Variables in top model are in bold and italic if base model is >2 **Δ**AIC from top model.

Testing for interaction of the binary predictors with the trend variable, the model without any interactions emerged as the top model. All interaction models had similar support to the main effect model but all interaction effects were weak (Table 3a).

### 3.3 Incidence of small and large individuals

Based on the 25th percentile of all weight measures, we classified individuals <=0.33 kg as small. Protection was the only important main effect, negatively affecting the baseline probability to be small (Table 3b). Under this main effect model, the trend in probability of being small was close to 0 (annual trend on the logit scale: −0.032, SE 0.040), i.e., there was no evidence suggesting a change in incidence of small individuals in the metapopulation over time. The best main effects model had more AIC support than any of the interaction models (Table 3b). As expected because of sexual dimorphism, females had a higher probability of being small than males (*β*: 0.507, SE 0.176).

Based on the 75th percentile of all weight measures, we classified individuals >=0.97 kg as large. Only 2 large individuals were ever captured at exploited sites (Figure 3) and we therefore removed exploitation as a predictor for the probability of being large.

The presence of hotels was the most important (positive) predictor of the baseline incidence of large individuals; the model with protection had similar support and a similarly strong positive effect but was similar in AIC to the null model and we therefore only retained the main effect of hotel (Table 3c). The trend in incidence of large individuals under the main effects model was close to 0 (0.057, SE 0.043), providing no evidence that the incidence of large individuals changed over time in the metapopulation. Neither of the two binary predictors showed important interactions with trend. As expected, females had a lower probability of being large (*β* = −3.579, SE 0.340).

### 3.4 Proportion of females

None of the three binary predictors (protection, exploitation, agriculture) were important as a main effect on baseline proportion of females in the 13 sites included in the analysis (Table 4). Under the base trend model, the annual trend in the proportion of females was close to 0 (−0.035, SE 0.032), suggesting that the proportion of females remained stable over the course of the study at the level of the metapopulation.

**TABLE 4:** Model selection of binary site‐level predictors of baseline (Effect = Main) and annual trend (Effect = Interaction) in proportion of females in the population.

Effect	Variable	ΔAIC	Beta	SE
Main	Exploited	0	−0.367	0.228
Main	NULL	0.18	/	/
Main	Agriculture	1.9	0.102	0.195
Main	Protected	2.1	0.063	0.218
Interaction	***Exploited***	0	−0.152	0.069
Interaction	NULL	2.76	/	/
Interaction	Protected	3.81	0.048	0.049
Interaction	Agriculture	4.48	0.025	0.047

*Note*: The “Main” base model (Variable = NULL) only has an annual trend (plus a site‐level random intercept to account for study design); the “Interaction” base model is equal to the best main effects model. Variables in top model are in bold and italic if base model is >2 ΔAIC from top model.

**FIGURE 4:**
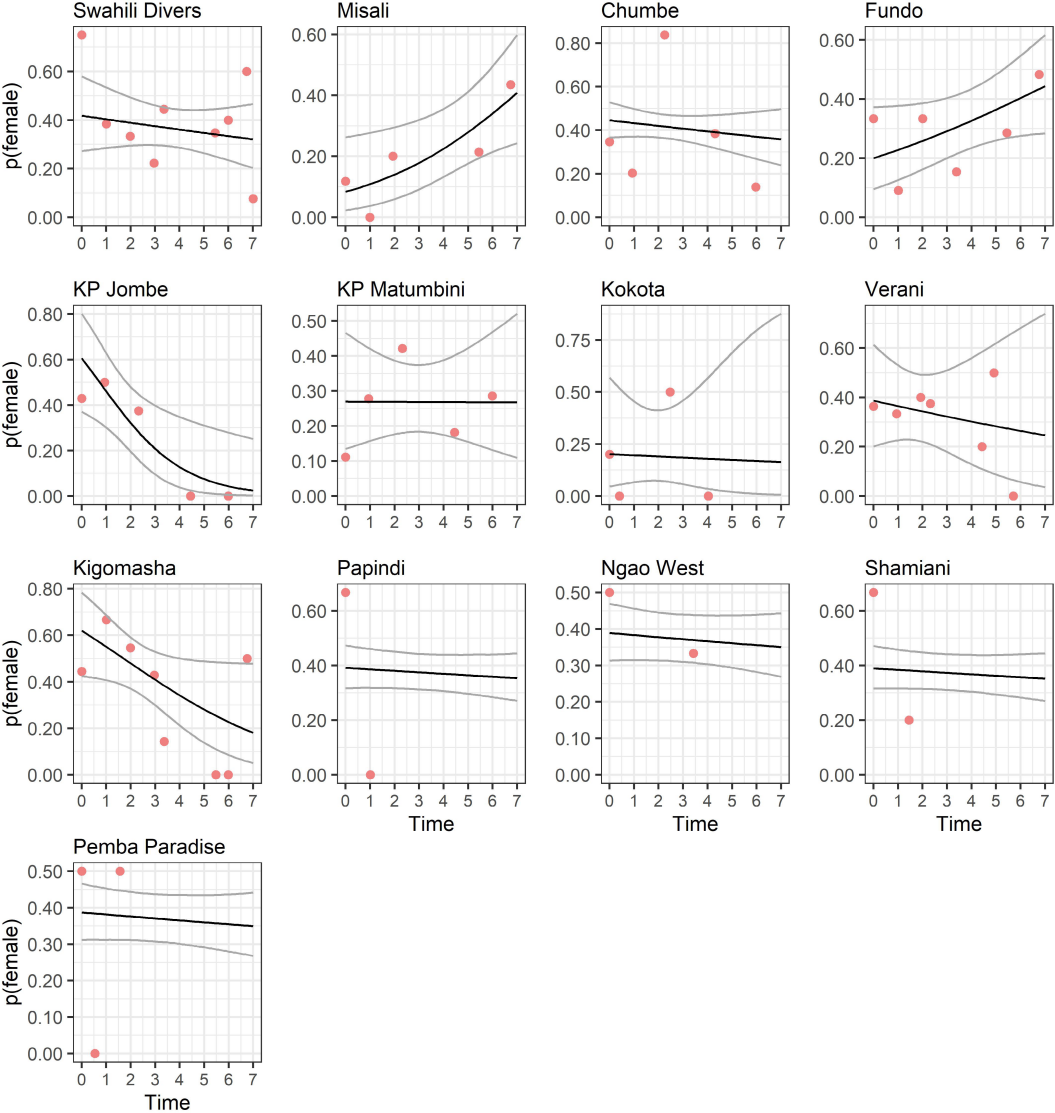
Proportion of females (out of all individuals caught) over time at 13 sites in/near Pemba (two surveyed sites were excluded because they only yielded captures during a single survey). Year 0 refers to the first year a site was surveyed, not a calendar year. Black lines show estimated trends in p (female) from a logistic regression; gray lines show 95% confidence intervals. For sites from Swahili Divers to Kigomasha, the model contained a fixed site specific intercept and trend; for remaining sites, the model contained a random site intercept, and the effect of exploitation (all unexploited) on trend. Red dots show proportion of females captured per survey (across multiple visits per survey) for all surveys to a site. Note the different scales of the y axis.

But exploitation interacted with the trend variable (Table 4), such that the proportion of females declined in exploited sites (trend: −0.175, SE 0.077) but remained stable in unexploited sites (trend: −0.023, SE 0.032; Figure 4). When estimating the proportions of females and their trends for each of the nine data‐rich subpopulations, that proportion was low (≤0.2) in the beginning of the study and increasing over time in two sites, high in the beginning (>0.6) and declining in two and mixed in the beginning (between 0.2 and 0.45) and stable in the remaining five sites (Figure 4, Table S4).

### 3.5 Additional information

We found no effect of surveys taking place before or after educational campaigns at the six sites for which such campaigns took place in September 2020 (*β*: −0.023, SE 0.182), which fits the fact that these sites had differing population trends (Figure 2). Six crabs were removed from KP Jombe in 2020, 8 in 2021, and 10 in 2022 (Figure 2). Against all caveats mentioned in the Methods, CPUE from student projects on Misali and Chumbe suggested that between 1999 and 2011 the crab population on Chumbe may have been increasing, whereas it may have been declining on Misali between 2006 and 2018 (Figure S11).

## 4 DISCUSSION

We found that the metapopulation of Zanzibari coconut crabs in sites sampled on and around Pemba, and on Chumbe Island, Unguja, has remained stable over the last 7 years, both in terms of numbers and sex and size/age composition. But some divergent trends were apparent at the subpopulation level for population size and the proportion of females, indicating spatial variation in population dynamics in this metapopulation. Identifying anthropogenic drivers of such divergent trends was hampered by small sample size, but results indicated that protection from anthropogenic impacts clearly benefitted crab populations.

### 4.1 Population trends

Previous studies using a subset of data analyzed here have demonstrated the negative impact of anthropogenic pressure, and the positive impact of formal protection, on static crab population size and weight (Caro et al., 2021, 2024). This is the first study to provide evidence that protection also seems to allow for subpopulation growth. This should be taken cautiously as there were only two protected populations in our sample, thus, we may be seeing site‐specific effects, not generalizable effects of protection. Nonetheless, this finding fits with what is known with coconut crab populations more broadly. Although we are not aware of other published studies linking coconut crab population trends to anthropogenic variables, Appoo et al. (2021) reported a large (ca. 5000 individuals) protected population in the Seychelles to be stable; and range‐wide spatial patterns of large populations remaining in largely undisturbed (by humans) islands (Cumberlidge et al., 2022) further support the notion that protection from human activity is important for maintaining crab populations (but see Helagi et al., 2015 for an example of an exploited population increasing in numbers).

Looking at specific data‐rich subpopulations, three showed increasing population trends (Swahili Divers, Misali, Chumbe). The latter two are formally protected and in Chumbe, sampling takes place near a hotel area, where crabs are known to feed on kitchen scraps and are viewed as a tourist asset. At Swahili Divers, crabs have to contend with nearby agriculture and human settlements but a hotel there provides food subsidies, as well as informal protection to crabs, as they are a tourist attraction (Caro et al., 2021). Four subpopulations appeared stable (Fundo, KP Jombe, KP Matumbini, Kokota), including the two sites in the dataset exposed to exploitation, and the site with known removals by the Department of Forestry. Meanwhile, populations at two likely non‐exploited sites were declining (Verani, Kigomasha), one of which is a hotel site although the hotel at Verani is only partially running. These site‐specific results highlight that crab population trends cannot readily be explained by single factors and are likely a product of a complex set of environmental (and possibly intrinsic) conditions, which are difficult to tease apart in the absence of large sample sizes. With respect to sustainable development of the local economy, understanding the role that hotels could play in preserving coconut crabs would be beneficial. The time series from other hotel sites in our dataset (Pemba Paradise, Shamiani) is still too short to be conclusive; one appears to have declined, while one fluctuated. An earlier study of many of the same sites suggested that crabs are larger around tourist facilities which may either indicate abundant and reliable foraging opportunities or attaining a greater age or both (Caro et al., 2021). Nonetheless, at a broader spatial scale, presence of crabs at the *shehia* level on Unguja was associated with absence of hotels (Hamad et al., 2023). As an aside, an earlier study at some of the same sites (Caro et al., 2021) reported different population trends than what we reported here (increasing subpopulations at KP Jombe and KP Matumbini, stable subpopulations at Swahili Divers, Kigomasha and Fundo, and declines at Verani, Misali and Chumbe), but this was based on only 2–3 years of data, highlighting the real necessity for long‐term monitoring to disentangle trends from short‐term fluctuations.

Our results of a stable metapopulation and some growing subpopulations are encouraging for the status of the coconut crab in/near Pemba, which is one of the species' strongholds in the West Indian Ocean (Caro et al., 2024). But it is important to remember that we included data from only 15 of 29 sites visited over the course of the study because they had sufficient populations to warrant repeated surveys. The remaining 14 ostensibly suitable sites (surveyed only once) had no or very few crabs, likely as a result of past population declines. By ignoring these sites, our assessment of the metapopulation as stable may be overly optimistic as, potentially, our survey here does not capture the loss of very small populations from the metapopulation. Indeed, all the subpopulations that we sampled were fairly large (>50 individuals), except Verani estimated at about 26 individuals (Caro et al., 2024).

### 4.2 Size, age and sex ratio

Size‐ (and consequently, male‐) biased harvest in coconut crabs has been shown to decrease average size, skew the size distribution toward smaller/younger individuals (Caro et al., 2021; Sato & Yoseda, 2010; Widiyanti et al., 2016), and skew sex ratio toward females (Sato & Yoseda, 2010; Yorisue et al., 2020). Our study corroborated previous findings, with crabs in formally protected sites being, on average, heavier, and populations showing a higher incidence of large and a lower incidence of small individuals, whereas crabs were, on average, lighter in exploited sites. Only two large individuals were ever found at exploited sites, further corroborating these patterns. A loss of large individuals could affect recruitment across the whole metapopulation, especially since older larger individuals disproportionately contribute to recruitment (Sato & Suzuki, 2010). However, there were no trends in weights or incidence of large or small individuals at the metapopulation or the subpopulation level, indicating populations remained largely stable in their size composition. Thus, our predictions about ongoing decline in average weight or large individuals due to exploitation and/or habitat loss at some subpopulation sites during our study were not supported. We speculate that rather than experiencing size‐biased harvest, crabs are harassed and killed by children for fun without regard to crab weight. Consistent with the constant prevalence of large individuals, we also found no evidence for our prediction of reduced recruitment (i.e., a decline in small individuals) across the metapopulation.

In addition to our analyses, we visually inspected the incidence of very small individuals (in the lower 5th percentile, sample size was too small for formal analysis) at data rich sites, but we found no evidence of patterns that would suggest synchronous recruitment events across sites (Figure S12).

Although the metapopulation was biased toward males (as are many populations of coconut crabs, e.g., Appoo et al., 2021; Rosnawati, 2020), analysis of the full dataset suggested that in exploited sites, the proportion of females declined over time. Similar to the effect of protection on population trends, however, this result should be interpreted with care, as there were only two exploited sites in our dataset. Indeed, analysis of data‐rich sites suggested that this result was driven by KP Jombe, where the proportion of females declined from 0.6 in 2017 to 0 in 2023. Whether that was due to exploitation or other local factors we cannot say. Certainly, offtake of 24 crabs over a three‐year period could have had an effect on the subpopulation sex ratio but we do not know the sex of individuals removed. In general, site‐level trends suggested that sites with few females at the beginning of the study had increasing proportions over time, whereas sites with many females had declining proportions but we cannot explain this possible cyclicity.

Finally, there appeared to be no patterns between weights, prevalence of small/large individuals, or trends in sex ratio on one hand, and population trends on the other (based on visual comparison of results for the different measures). Thus, our data provide no clues as to which demographic processes more likely underlie the diverging population trends. The very low incidence of small individuals in the growing population of Chumbe is noteworthy, as a population cannot grow without recruitment. Sampling in Chumbe focused on the hotel kitchen area, but crabs also occur in other parts of the island (though at lower density, Caro et al., 2024). It is possible that there is some spatial separation of large old individuals and new recruits, perhaps because smaller animals avoid confrontation with larger individuals who monopolize the resource rich kitchen area. This could lead to a bias in sampling toward larger individuals. In 2022, crabs were also captured in two adjacent areas on Chumbe (data not used for trend analysis). Their weights suggest that crabs outside the kitchen area may indeed be smaller, on average (median 0.95 kg, *n* = 38), than those captured in the kitchen area (median 1.4 kg, *n* = 93), but that difference was not statistically significant (Wilcoxon test *p*‐value .15).

We relied on prevalence of large and small individuals to study trends in recruitment and adult mortality. Ideally, processes underlying population trends, such as survival and recruitment, should be studied directly. In spite of efforts to document individuals photographically so they could be identified across surveys, and high effort investment in repeated surveys at some sites (e.g., Chumbe), recaptures of individuals across surveys were exceedingly rare, prohibiting estimation of survival. Given the consistent presence of heavy individuals at these locations, it is unlikely that this is a result of very low adult survival. More likely, this is a function of movement out of the surveyed sites (in cases where these are not contained; though recaptures were also rare at “contained” surveyed sites like Swahili Divers), or of cryptic behavior (hiding below ground), the same mechanisms that may lead to the high variability in captures among nightly visits (see below). Recruitment is challenging to study directly owing to the small size and (likely) large number of newly terrestrial coconut crabs carrying mollusk shells, which are not typically sampled in surveys of adults. Recruitment into the catchable (non‐shell) population could be estimated with open population CMR models, but this, again, is hindered by the lack of recaptures across surveys. Thus, a better understanding of crab movement behavior and monitoring protocols adapted to this behavior is likely the key to studying demographic rates in this species.

### 4.3 Implications for metapopulation monitoring

Monitoring subpopulations comprising a metapopulation over space and time is inherently difficult for any species (e.g., McIntosh et al., 2018). With limited personnel and financial resources that bedevil most conservation projects, a constant trade‐off develops between the number of sites monitored and the amount of time spent visiting each site, both within and across seasons. Overall, number of sites that we could monitor repeatedly across years was limited to less than 20 and not all of these could be reached every year. Our goal of assessing spatial drivers of trends would have benefitted from sampling more sites (though in the focal metapopulation, the number of available sites is limited), whereas studies mainly interested in temporal drivers of population size such as climate conditions would likely require revisiting sites more consistently over time.

The ability to detect trends is further affected by within‐season variability of population measures (Hatch, 2003; Seavy & Reynolds, 2007). The large amounts of unexplained night‐to‐night variation in capture numbers in the present study requires repeated visits per survey to adequately characterize survey‐specific population status, but several sites were only visited once per survey. For instance, removals from the KP Jombe site may have contributed to apparent decline or fluctuations in CPUE, but data from the limited number of single‐visit surveys are too sparse to detect such effects. Only moon phase came out as important in explaining this nightly variation, even though rain/humidity has been cited as being conducive to capturing crabs (pers comm to TC). Previous studies have noted the extreme short‐term fluctuations in capture numbers as well as catch composition with respect to sex and size (Appoo et al., 2021) and have also noted the importance of moon phase for coconut crab activity. To better separate signal (trend) from noise in crab capture time series and thus create more efficient monitoring programs, we may need to understand crab movement behavior better. In particular, extremely low numbers of recaptures of this long‐lived species within and between seasons demands explanation. Individuals apparently spend long periods underground reappearing only infrequently according to as yet unknown phenotypic or ecological factors. The lack of associations between above ground activity and three measures of precipitation are difficult to explain given the importance of water for drinking and avoiding desiccation in this terrestrial species. Possibly crabs respond to extremely local events at their refuge locations rather than at the *shehia* level; rainfall on Zanzibar can be very localized.

The lack of recaptures within seasons also precludes an analysis that formally accounts for imperfect and varying detection, for example, with capture‐mark‐recapture (CMR) models. Although we account for major sources of variation in detectability of crabs—using effort as an offset, by including moon phase as a visit‐level variable—our analysis assumes that the remaining variation in survey‐level CPUE (both in space and time) is due to variation in abundance. Without recaptures to estimate detection probability, we cannot test whether this assumption is met, or whether some of that variability is indeed due to variation in detection (e.g., Pollock et al., 2002). Caro et al. (2024) developed an integrated model that combined CMR data from sites with some recaptures with count data from other surveys to estimate abundance. Data suitable for CMR analysis, however, came from five sites only, and were limited to post‐2017 surveys. Thus, to characterize detectability in space and time, as would have been necessary in our analysis of population dynamics, this integrated approach was not an option.

Furthermore, the scale at which subpopulations can be sampled is often limited. In our study, aside from Swahili Divers, Verani and Kigomasha which are “contained” populations in that there are negligible numbers outside the sampled area, other sites were geographically more extensive, but we only sampled limited areas within those sites. We already mentioned the possibility of the sampling in the hotel area on Chumbe biasing the sample toward large individuals. Similarly, spatially restricted and non‐representative sampling at this and other sites could yield biased estimates of population trends, should these vary in space at the local scale, as has been shown for density (Caro et al., 2024 for Chumbe and Misali) and size (Rosnawati, 2020; this study). Thus, ideally, sampling would include spatial replication within geographically more extensive sites.

Finally, the time period over which the subpopulations here were sampled was relatively short, which is common in spatially replicated monitoring projects owing to their logistic and financial difficulties (Ojanen et al., 2013). Short time series limit our ability to detect temporal patterns; for example, we suspect that not enough time has elapsed to see possible effects of the 2020 education campaign. We tried to extend the time window over which crabs were monitored at two sites, Chumbe and Misali, but were unsuccessful owing to different methodologies (different methods of finding crabs, different measuring techniques, different sampling areas, etc.) both among these studies themselves and in relation to our study. It points to the vital importance of adhering to standardized methodology, proper reporting of study design, and to training subsequent researchers.

### 4.4 Conservation implications

This medium‐term study of a metapopulation of coconut crabs shows that subpopulation trends are highly variable but that formal Government protection appears to benefit subpopulation growth. In essence more fully protected areas need to be set aside now, and currently protected areas need to be managed properly, to maintain this species on the Zanzibar archipelago. On Pemba alone, one of the current two protected sites, Misali, has been leased for hotel development; areas have been delineated on KP Jombe and KP Matumbini for development (all part of the ZIPA program https://www.zipa.go.tz/services/small‐islands/), and a hotel is being built at Kigomasha. This is a very serious cause for concern for Zanzibar's natural capital. Hotels may be less detrimental than other forms of development if owners want to foster ecological stewardship and set aside coastal forest habitat within their boundaries for tourists, but they are environmentally detrimental during the building phase. Many hoteliers simply do not take wildlife into account and legal restrictions are rarely adhered to so conservationists and the Government need to work with lease holders to promote good conservation stewardship on Zanzibar's small islands.

## AUTHOR CONTRIBUTIONS

**Rahel Sollmann:** Conceptualization (equal); data curation (equal); formal analysis (lead); funding acquisition (supporting); investigation (equal); methodology (equal); project administration (supporting); resources (supporting); software (lead); supervision (equal); validation (equal); visualization (equal); writing – original draft (lead); writing – review and editing (equal). **Tim Caro:** Conceptualization (equal); data curation (equal); formal analysis (supporting); funding acquisition (lead); investigation (equal); methodology (equal); project administration (lead); resources (equal); software (supporting); supervision (equal); validation (equal); visualization (equal); writing – original draft (equal); writing – review and editing (equal).

## CONFLICT OF INTEREST STATEMENT

The authors declare no competing interests.

## Supporting information


Data S1.

## Data Availability

All data and scripts used for the analyses are available on github at: https://zenodo.org/doi/10.5281/zenodo.12755505
