# Supplementary material for: Spatio‐temporal metapopulation trends: The coconut crabs of Zanzibar
Source: Ecol Evol. 2024 Aug 27;14(8):e70168. doi: 10.1002/ece3.70168 (PMC11349606; doi:10.1002/ece3.70168)
Supplement: Supplementary file 1 — Data S1. [file ECE3-14-e70168-s001.pdf]

Sollmann, R., and Caro, T.

Spatio-temporal metapopulation trends: the coconut crabs of Zanzibar

Ecology and Evolution

Supporting Information S1: Additional analyses and results

Table S1: Selection of the best effort measure to use as an offset in a Poisson model of number of coconut crabs caught (CPUE) fit to nightly capture data from 15 sites on/near Pemba collected between 2016 and 2023.

**Effort**	**ΔAIC**	**Definition**
Infield	0	Time in field during visit (in minutes)
ActualSPmin	161.13	Total time every person on search team spent searching (in minutes)
SearchEst	201.5	Time in field minus time measuring captured crabs (minutes)

Table S2: Selection of visit-level covariates affecting coconut crab capture rates. Moon.bin = binary moon phase (1 = 12-16, i.e., near/full moon, and 0 = all other moon phases), RainDay.sc = precipitation the day of the visit (in mm, scaled), RainWeek.sc = total precipitation in the week preceding the visit (in mm, scaled), Rain.bin = binary precipitation (yes or no) on the day of the visit. Based on Poisson mixed model fit to nightly capture data from 15 sites on/near Pemba collected between 2016 and 2023.

**Variable**	**ΔAIC**	**beta**	**SE**
Moon.bin	0	-0.222	0.120
RainWeek.sc	1.22	0.063	0.040
Null	1.60	/	/
RainDay.sc	1.95	0.052	0.040
Moon.	3.38	-0.025	0.053
Rain.bin	3.55	-0.019	0.080

Table S3: Estimates of site-specific annual population rates of change for coconut crabs (with standard error SE and 95% Wald confidence interval limits, CI.lower and CI.upper) for 9 data-rich sites in/near Pemba, based on annual trend estimates from a Poisson mixed model with fixed site-specific intercept and trend.

**Site**	**Rate of change**	**SE**	**CI.lower**	**CI.upper**	**Category**
Swahili Divers	1.12	0.06	1	1.25	increasing
Misali	1.1	0.08	0.96	1.26	increasing
Chumbe	1.19	0.08	1.04	1.36	increasing
Fundo	0.98	0.07	0.85	1.14	stable
KP Jombe	0.94	0.09	0.77	1.14	stable
KP Matumbini	0.95	0.08	0.8	1.13	stable
Kokota	1.03	0.17	0.74	1.43	stable
Verani	0.87	0.1	0.7	1.09	declining
Kigomasha	0.79	0.07	0.67	0.93	declining

Table S4: Starting proportion of females in the first survey (P) and linear trend (on the logit scale) over time in nine coconut crab populations on/near Pemba. Trend estimates < -0.1 are considered declining, and > 0.1 increasing. Estimates from a logistic regression with fixed site-specific intercept and trend.

**Site**	**P**	**Trend**	**SE**	**CI.lower**	**CI.upper**	**Category**
Fundo	0.2	0.17	0.09	-0.01	0.35	increasing
Misali	0.08	0.29	0.12	0.04	0.53	increasing
Swahili Divers	0.42	-0.06	0.08	-0.21	0.09	stable
Verani	0.39	-0.09	0.2	-0.49	0.3	stable
KP Matumbini	0.27	0	0.12	-0.24	0.24	stable
Chumbe	0.45	-0.05	0.06	-0.16	0.06	stable
Kokota	0.2	-0.04	0.34	-0.7	0.63	stable
Kigomasha	0.62	-0.29	0.15	-0.57	0	declining
KP Jombe	0.61	-0.59	0.24	-1.05	-0.13	declining

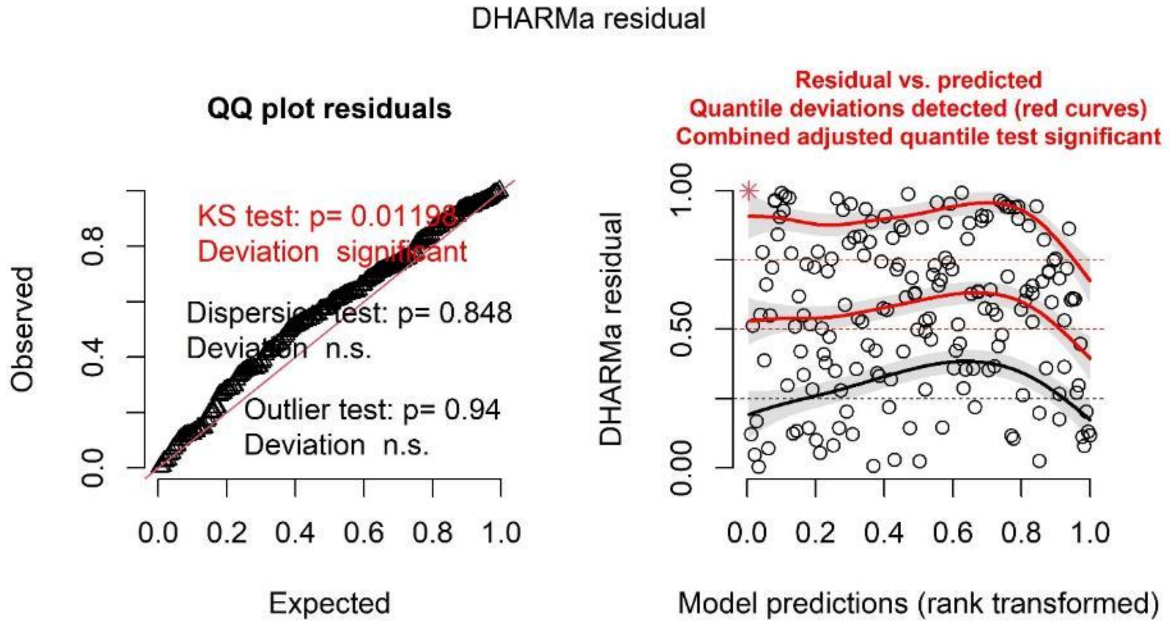

Figure S1: Residual plot for best Poisson mixed model (with effort as offset, site random effect, survey random effect nested within site, agriculture as main effect and protection interacting with annual trend) fit to nightly coconut crab capture data from 15 sites in/near Pemba.

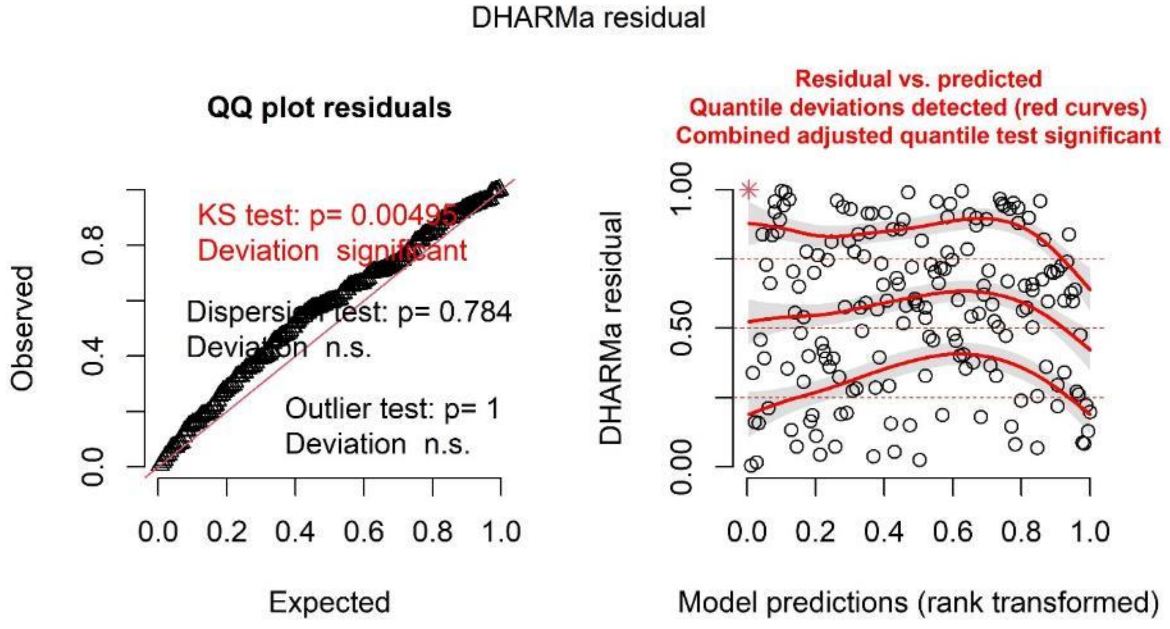

Figure S2: Residual plot for same model as Figure S1, but using a negative binomial instead of a Poisson distribution (the dispersion parameter was >20, suggesting no overdispersion).

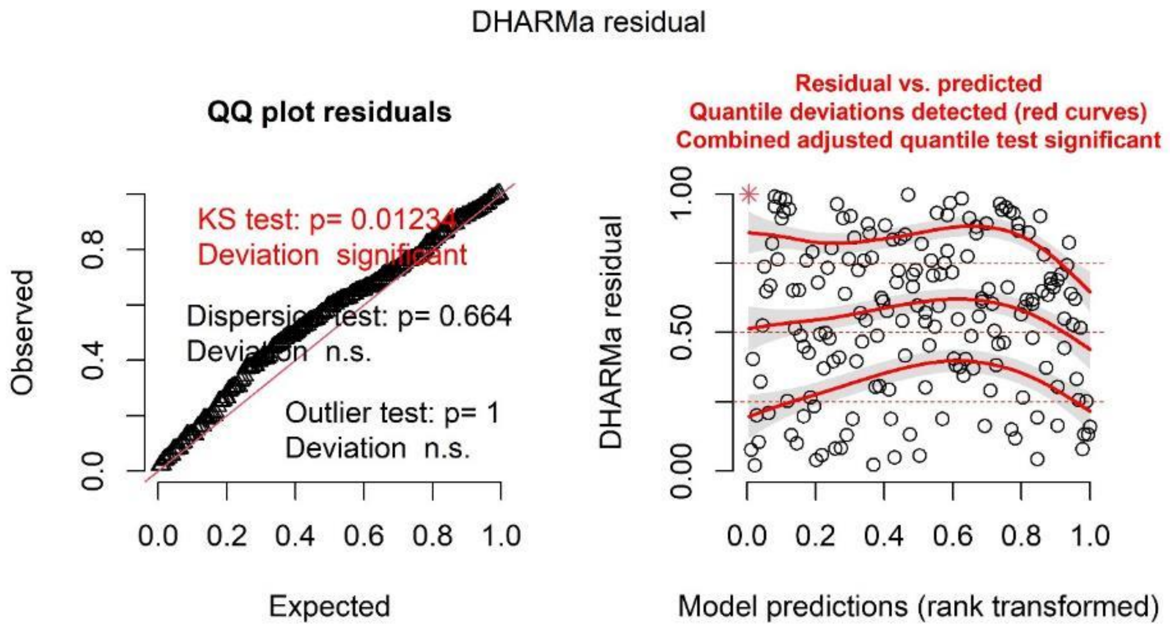

Figure S3: Residual plot for same model as in Figure S1, but adding a visit-level random effect to account for extra variability.

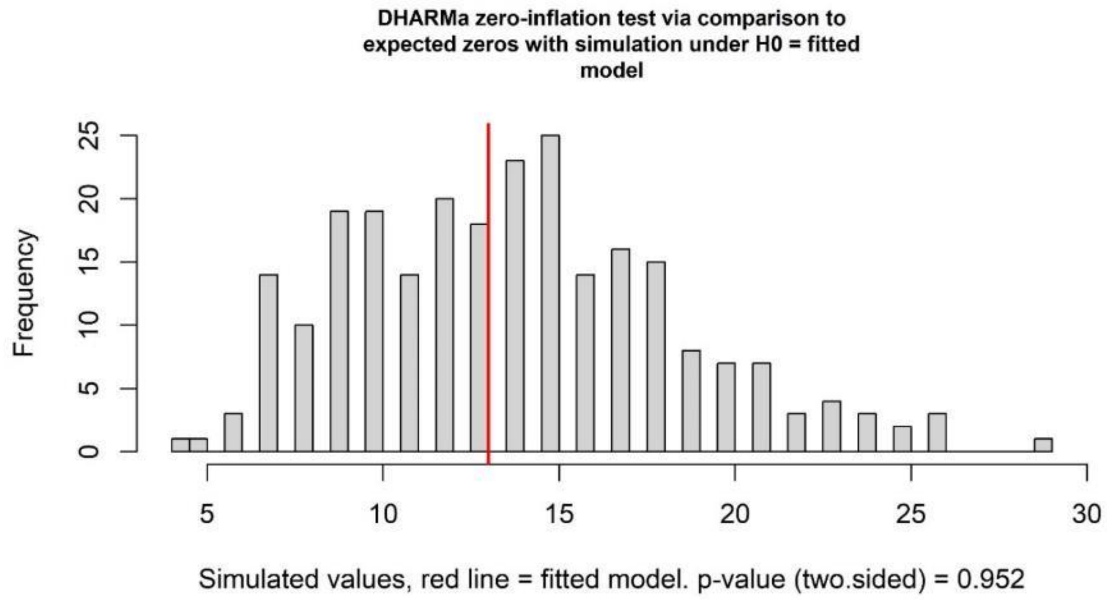

Figure S4: DHARMa test for zero-inflation for model described under Figure S1. No indication for zero-inflation in the data.

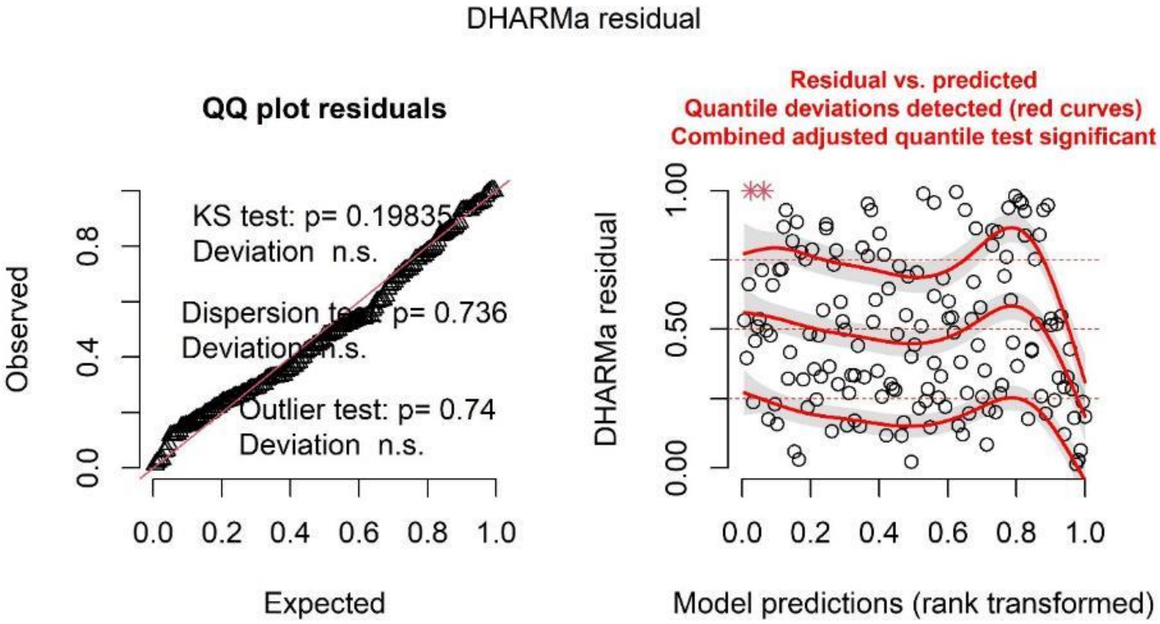

Figure S5: Residual plot for Poisson mixed model (with effort as offset, survey random effect nested within site, and fixed site-specific intercepts and trends) fit to nightly coconut crab capture data from 9 data-rich sites in/near Pemba.

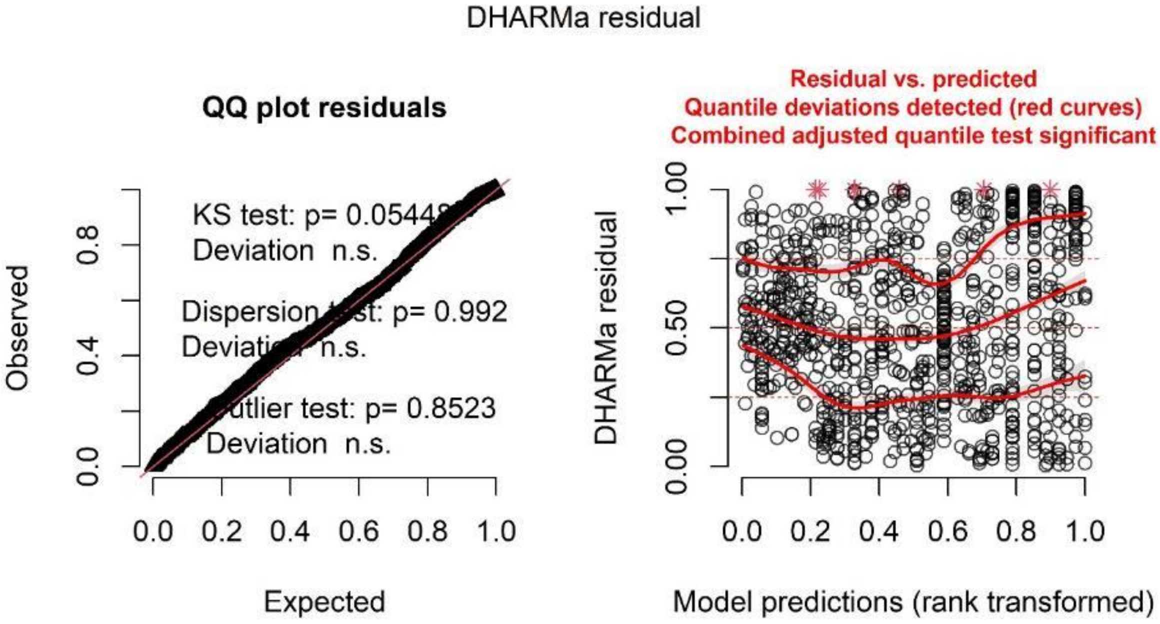

Figure S6: Residual plot for linear mixed model (with random site-specific intercept, sex, protection and exploitation as fixed main effects, and an annual trend) fit to square-root transformed weights (in kg) of coconut crabs from 13 sites in/near Pemba.

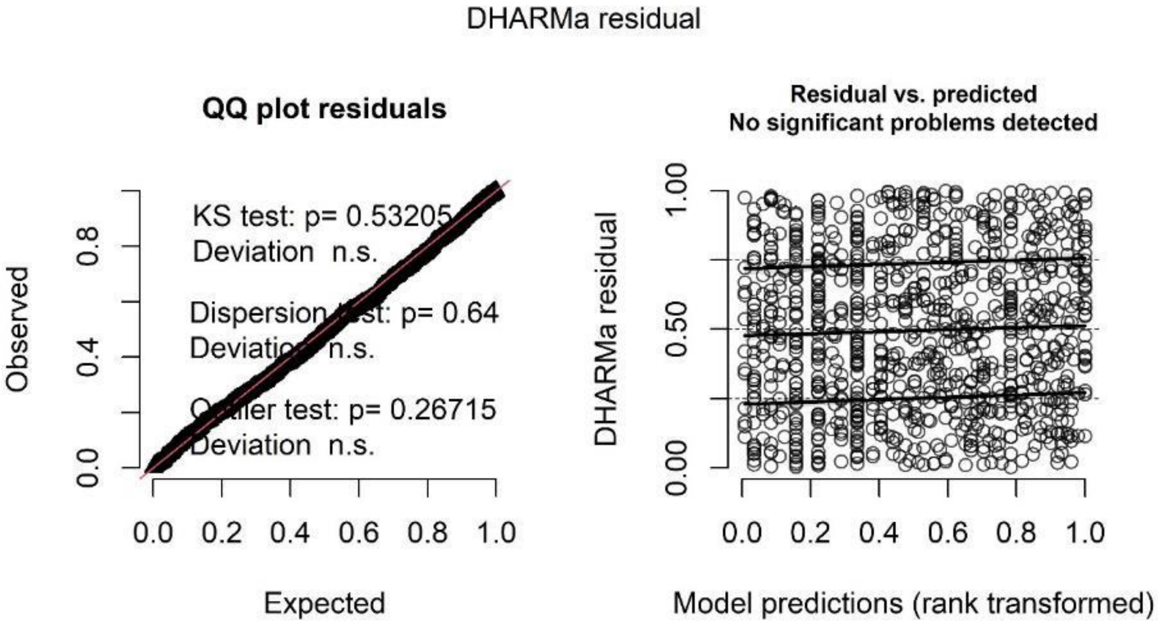

Figure S7: Residual plot for mixed logistic regression (with random site-specific intercept, sex and protection as fixed main effects, and an annual trend) fit to binary response indicating whether an individual was small (<=0.33kg) or not for coconut crabs from 13 sites in/near Pemba.

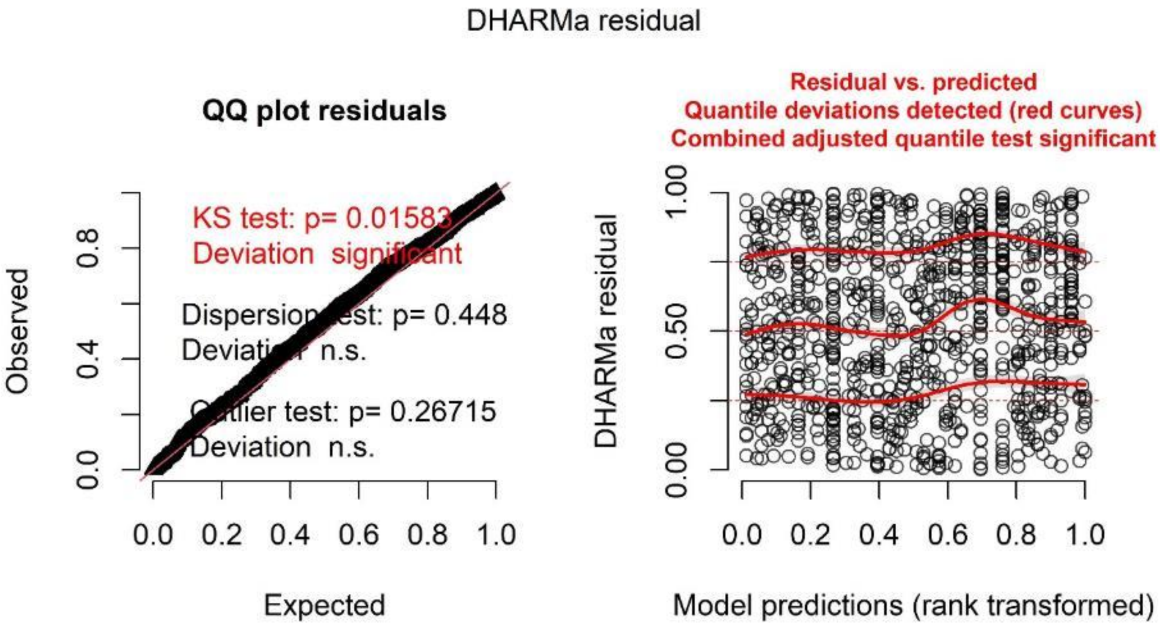

Figure S8: Residual plot for mixed logistic regression (with random site-specific intercept, sex and hotel as fixed main effects, and an annual trend) fit to binary response indicating whether an individual was large (>=0.97 kg) or not for coconut crabs from 13 sites in/near Pemba.

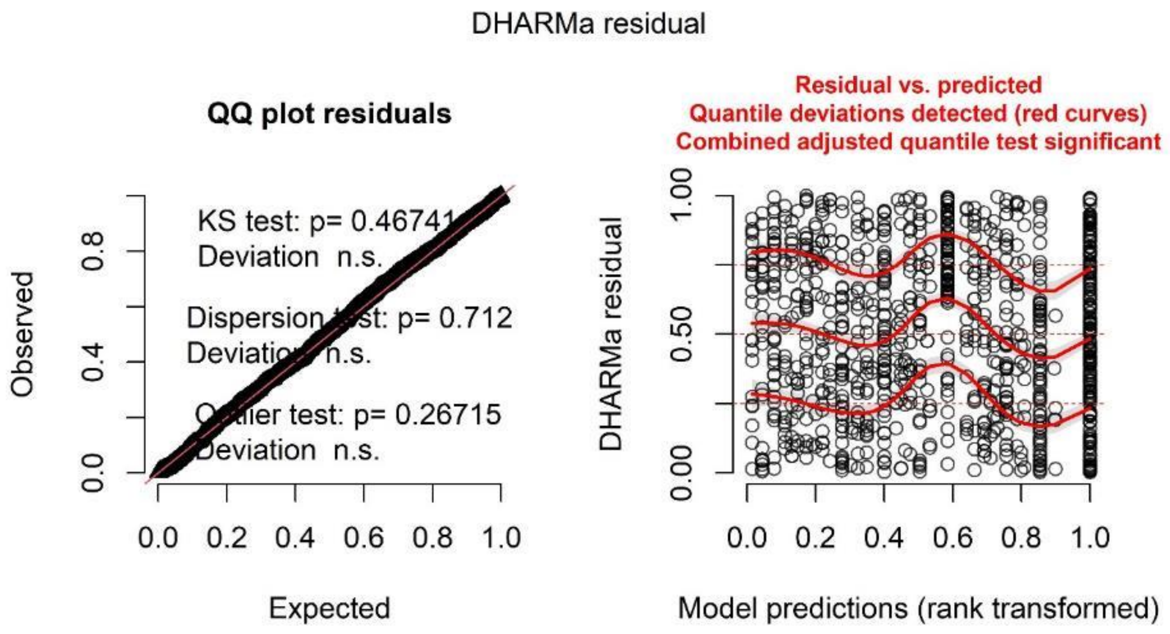

Figure S9: Residual plot for mixed logistic regression (with random site-specific intercept, and exploitation interacting with an annual trend) fit to binary response indicating whether an individual was female or not or not for coconut crabs from 13 sites in/near Pemba.

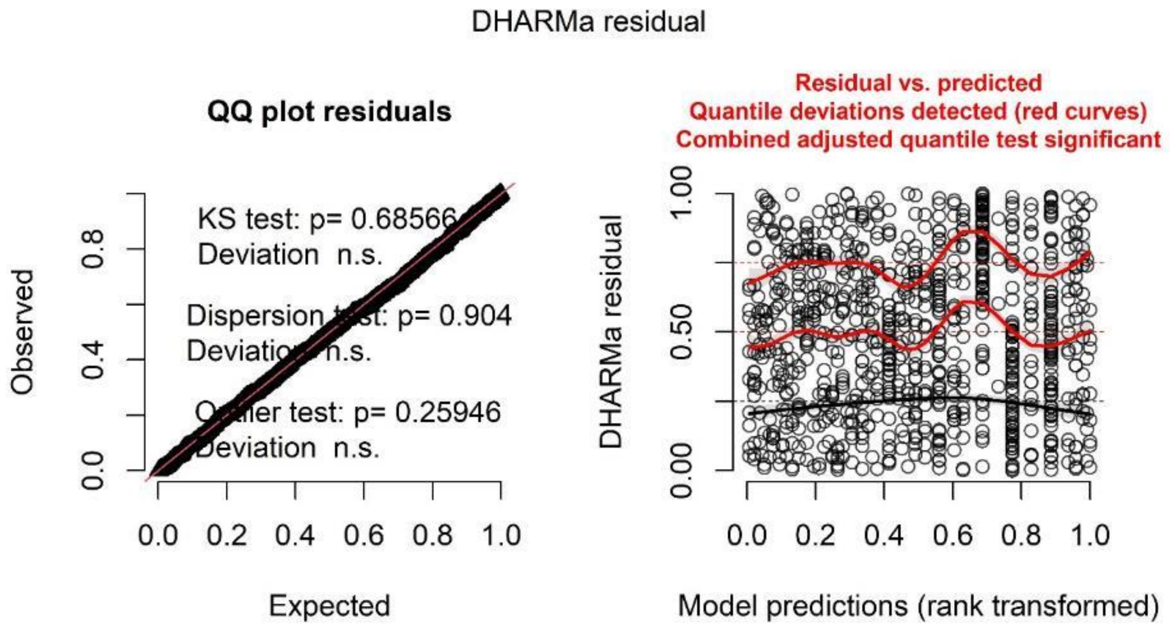

Figure S10: Residual plot for logistic regression (with fixed site-specific intercept and trend) fit to binary response indicating whether an individual was female or not or not for coconut crabs from 9 data-rich sites in/near Pemba.

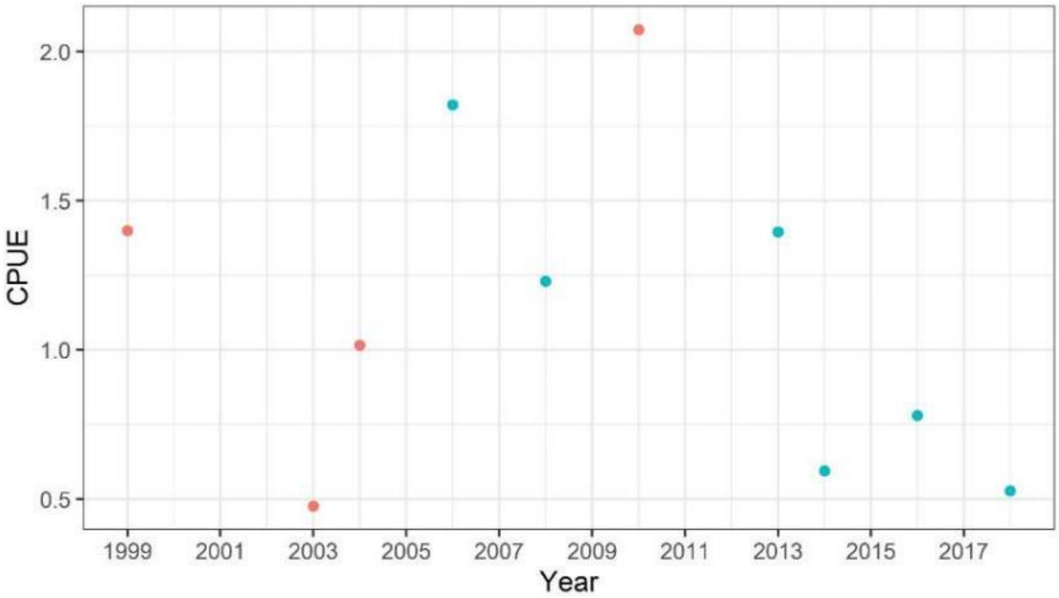

Figure S11: Overall catch per unit effort (crabs captured divided by number of locations surveyed) for student projects on the islands of Chumbe (red) and Misali (blue). Note that CPUE from this plot cannot be compared to CPUE obtained in the present study as detailed effort information was not available for these student projects (data obtained from School for International Training unpublished reports).

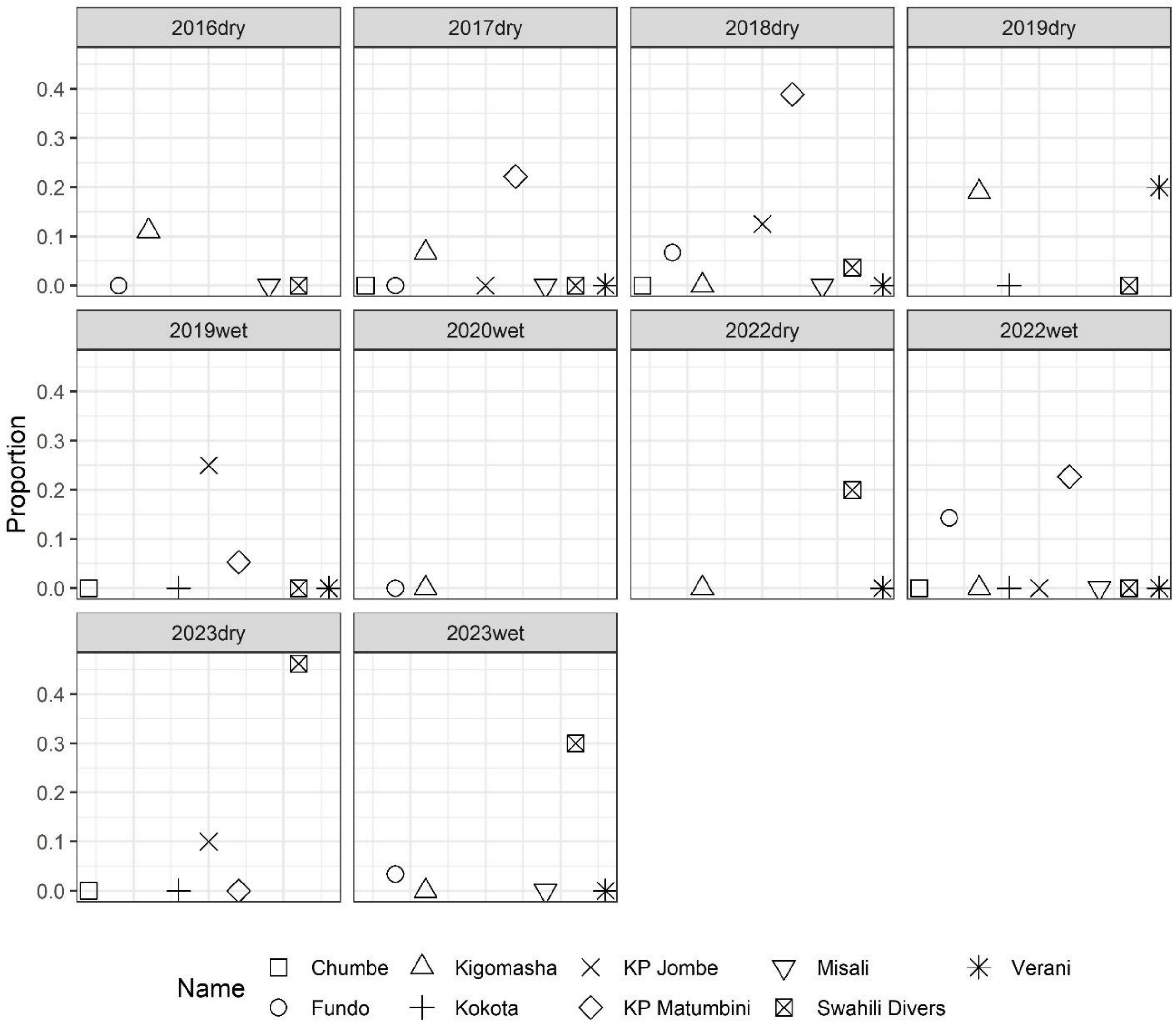

Figure S12: Proportion of very small coconut crabs (<=0.14kg) caught per survey (year-season combination) across nine data-rich sites in/near the Pemba archipelago, Zanzibar. Missing symbols indicate that site was not sampled in a particular survey.
